# Distinct cortical and subcortical predictors of Purdue Pegboard decline in Parkinson’s disease and atypical parkinsonism

**DOI:** 10.1038/s41531-023-00521-0

**Published:** 2023-06-05

**Authors:** Bradley J. Wilkes, Emily R. Tobin, David J. Arpin, Wei-en Wang, Michael S. Okun, Michael S. Jaffee, Nikolaus R. McFarland, Daniel M. Corcos, David E. Vaillancourt

**Affiliations:** 1 Department of Applied Physiology and Kinesiology, University of Florida, Gainesville, FL USA; 2 Department of Neurology, Norman Fixel Institute for Neurological Diseases, University of Florida, Gainesville, FL USA; 3 Department of Physical Therapy and Human Movement Sciences, Northwestern University Feinberg School of Medicine, Chicago, IL USA; 4 Department of Biomedical Engineering, University of Florida, Gainesville, FL USA

**Keywords:** Motor control, Parkinson's disease

## Abstract

Objective measures of disease progression are critically needed in research on Parkinson’s disease (PD) and atypical Parkinsonism but may be hindered by both practicality and cost. The Purdue Pegboard Test (PPT) is objective, has high test-retest reliability, and has a low cost. The goals of this study were to determine: (1) longitudinal changes in PPT in a multisite cohort of patients with PD, atypical Parkinsonism, and healthy controls; (2) whether PPT performance reflects brain pathology revealed by neuroimaging; (3) quantify kinematic deficits shown by PD patients during PPT. Parkinsonian patients showed a decline in PPT performance that correlated with motor symptom progression, which was not seen in controls. Neuroimaging measures from basal ganglia were significant predictors of PPT performance in PD, whereas cortical, basal ganglia, and cerebellar regions were predictors for atypical Parkinsonism. Accelerometry in a subset of PD patients showed a diminished range of acceleration and irregular patterns of acceleration, which correlated with PPT scores.

## Introduction

Parkinson’s disease (PD) is a debilitating disorder characterized by both motor and non-motor manifestations and is the second most common neurodegenerative disease. A major hurdle in PD is the scarcity of objective and reliable measures to assess the effectiveness of treatments potentially altering disease progression^1^. Moreover, related Parkinsonian syndromes, including multiple system atrophy (MSA) and progressive supranuclear palsy (PSP), present similarly to PD in early disease stages but have more advanced rates of decline and distinct pathological profiles. Currently, the common measures used to characterize motor disease progression in PD and atypical Parkinsonian disorders are subjective in nature, such as the Movement Disorders Society Unified Parkinson’s Disease Rating Scale (MDS-UPDRS). Although objective measures of dopamine transporter function, such as single photon emission computed tomography (DAT SPECT), free-water imaging, and positron emission tomography (F-DOPA PET), have shown good sensitivity and reliability in tracking disease progression in the first few years of being diagnosed with PD^2–5^, these assays can be time-consuming and expensive to implement on a wide scale and the DAT SPECT and F-DOPA PET can plateau after a few years post-diagnosis^6,7^. Phase 2 and Phase 3 clinical trials for interventions that slow disease progression in Parkinsonism can have several sites within one country^8,9^ or multiple international sites^10–12^. These studies can be costly to perform and challenging to standardize across sites due to factors such as the employment of novel pharmaceutical agents and advanced medical imaging techniques. Such challenges can hinder or prevent the inclusion of subjects in areas without access to adequate medical imaging facilities, which limits the generalizability of such findings. There remains a critical need for objective, reliable, inexpensive, and clinically feasible readouts for tracking disease progression in PD and atypical Parkinsonian disorders.

The Purdue Pegboard Test (PPT) is objective, inexpensive, and easy to administer with high test-retest reliability^13^. Moreover, there are a wealth of studies evaluating PPT performance in Parkinsonism and other conditions^14–17^, as well as normative data for healthy aging individuals^18^. Recently, two large studies of patients with PD found a longitudinal decline in PPT performance as disease severity increased. Hinkle and Pontone^19^ showed that baseline performance on the PPT was a significant predictor of declining psychomotor processing speed and detrimental changes in activities of daily living. Liu et al.^20^ showed a reliable rate of longitudinal decline in PPT performance in PD with a Rapid Eye Movement Behavior Disorder diagnosis. A shortcoming of both studies was the lack of a healthy control (HC) group, making it unclear whether declining performance on the PPT was specific to Parkinsonism or also impacted by aging. Other objective measures of motor function have been proposed for assessing progression effects in PD, such as the timed up and go test and nine hole peg test^21^. Although these assessments share many of the appealing qualities of the PPT, such as ease of administration and high test-retest reliability, standard implementation of those tasks is not sensitive to longitudinal progression effects in PD^22,23^.

Our first goal was to assess longitudinal changes in PPT performance in a large multisite cohort of patients with PD, MSA, and PSP compared to a similarly aged group of HCs. We hypothesized that Parkinsonian patients would show a significant decline in Purdue Pegboard performance after 1 year but that HCs would not. Specifically, we hypothesized that subjects with MSA and PSP would have lower PPT scores and more advanced rates of decline than PD and HC. We also hypothesized that longitudinal changes in Purdue Pegboard in Parkinsonian patients would correlate with worsening motor symptom severity measured by Part III of the Movement Disorders Society Unified Parkinson’s Disease Rating Scale (MDS-UPDRS-III).

Our second goal was to determine if the PPT score at 1-year follow-up could be predicted from baseline free-water imaging data of brain microstructure. We hypothesized that baseline free-water imaging data in key regions of neuromotor circuitry would provide robust estimates of PPT performance at 1-year follow-up and that PD and atypical parkinsonism would show distinct sets of disease-relevant predictors.

Our third goal was to evaluate accelerometry during the performance of the PPT to determine which kinematic features were different in patients with PD compared to HCs. The PPT is a repetitive goal-oriented task that requires accurate hand movements. Given that PD patients were shown to have a greater degree of a speed-accuracy trade-off than HCs^24^, we hypothesized that PD patients would show a diminished range of acceleration values, as measured by the standard deviation of acceleration. Given the repetitive nature of motor output expected to properly perform the PPT, we hypothesized that PD patients would show more irregular acceleration patterns compared to HCs, as measured by the approximate entropy of acceleration.

## Results

### Demographic and clinical data—longitudinal PPT

There was a significant difference in age [F(3, 275) = 4.018, *p* = 0.008] between PD, PSP, MSA, and HC subjects, which is summarized in Table 1. In a post hoc analysis, we found that PSP subjects had a significantly higher age compared to HC subjects (*p* = 0.027) and PD subjects (*p* = 0.004). We found that in HCs, there were no significant differences in age compared to MSA (*p* = 0.827) and PD (*p* = 0.827). Additionally, we found that MSA subjects did not have any significant differences in age compared to PD (*p* = 0.827) and PSP (*p* = 0.116).

**Table 1:** Participant demographic information—Purdue Pegboard test scores.

Demographic	PD (107M/57F)	PSP (17M/22F)	MSA (17M/6F)	HC (20M/33F)	*p*-value
Age	64.69 ± 8.129	69.62 ± 5.861	65.61 ± 8.631	65.17 ± 8.706	0.008**
Disease duration (years)	2.692 ± 2.349	2.932 ± 2.730	3.101 ± 2.667	-	0.685
**Visit 1**					
MOCA	26.35 ± 2.449	22.64 ± 4.196	22.52 ± 5.080	27.11 ± 1.888	<0.001***
MDS-UPDRS-III	29.92 ± 12.50	42.92 ± 15.77	49.04 ± 18.60	3.151 ± 2.663	<0.001**
**Visit 2**					
MOCA	26.55 ± 2.641	21.39 ± 5.133	20.96 ± 7.183	27.55 ± 1.957	<0.001***
MDS-UPDRS-III	33.03 ± 12.29	54.13 ± 14.05	57.39 ± 20.61	4.094 ± 3.353	<0.001***

Participant sex, site, age, and disease duration in years (mean ± 1 SD). Age and disease duration are based on the baseline visit. The Montreal Cognitive Assessment (MOCA) and Movement Disorders Society Unified Parkinson’s Disease Rating Scale Part III (MDS-UPDRS-III) for both baseline (Visit 1) and the 1-year follow-up (Visit 2) include mean ± 1 SD. One-way ANOVA was completed to see if all groups were similar or different from each other. Significance indicated by *p < 0.05, **p < 0.01, and ***p < 0.001.

We did not find any significant differences in disease duration between PD, PSP, and MSA [F(2,223) = 0.379, *p* = 0.685], which is summarized in Table 1. HCs were excluded since they do not have a disease duration. We did not find any significant differences in the disease duration of MSA compared to PD (*p* = 0.792) and PSP (*p* = 0.792). In addition, there was no significant difference (*p* = 0.792) in the disease duration of PD compared to PSP.

There was a significant main effect of diagnosis on Visit 1 Montreal Cognitive Assessment (MOCA) score [F(3,275) = 29.52, *p* < 0.001] and Visit 2 MOCA score [F(3,275) = 40.63, *p* < 0.001], which is summarized in Table 1. In the post hoc analysis, we found that HC had significantly higher MOCA scores at Visit 1 (*p* < 0.001) and Visit 2 (*p* < 0.001) compared to MSA and PSP subjects. Furthermore, we found that PD subjects had significantly higher MOCA scores at Visit 1 (*p* < 0.001) and Visit 2 (*p* < 0.001) compared to MSA and PSP subjects. There were no significant differences in MOCA score at Visit 1 (*p* = 0.126) nor Visit 2 (*p* = 0.094) between PD and HC. Additionally, there was no significant difference in MOCA score Visit 1 (*p* = 0.878) and Visit 2 (*p* = 0.647) between MSA and PSP.

For the MDS-UPDRS-III, there was a significant main effect of diagnosis on Visit 1 [F(3,275) = 111.5, *p* < 0.001] and Visit 2 [F(3,275) = 164.2, *p* < 0.001], summarized in Table 1. In the post hoc analysis, we found, as expected, HCs had significantly lower MDS-UPDRS-III compared to PD, MSA, and PSP in both Visit 1 and Visit 2 (*p* < 0.001). However, in both Visit 1 (*p* < 0.001) and Visit 2 (*p* < 0.001), PD subjects had a significantly higher MDS-UPDRS-III score compared to MSA and PSP. There was no significant difference in MDS-UPDRS-III score in Visit 1 (*p* = 0.074) and Visit 2 (*p* = 0.356) between MSA and PSP.

### Longitudinal changes in Purdue Pegboard

The PPT includes four tasks: dominant hand, non-dominant hand, both hands simultaneously, and assembly task. To evaluate progression effects on the PPT, we performed two-factor (2×4) repeated measures ANOVAs for each of the four tasks. Time (Visit 1, Visit 2) was a within-subjects factor, and diagnosis (PD, MSA, PSP, or HC) was a between-subjects factor. Age, testing site, Visit 1 MOCA, and sex were included as covariates. There was a significant main effect of diagnosis on each of the four tasks (*p* < 0.001). There was no significant main effect of time in any of the four tasks. There was a significant interaction between diagnosis and time for all tasks: the dominant hand task [F(3,271) = 4.725, *p* < 0.001], non-dominant hand task [F(3,271) = 5.783, *p* = 0.001], both hands task [F(3271) = 7.081, *p* < 0.001] and assembly task [F(3,271) = 4.383, *p* = 0.005] and are summarized in Table 2. In order to further evaluate the significant time by diagnosis interaction across the four groups, we calculated the 1-year change (PPT_visit2_ − PPT_visit1_) for each PPT task for each subject and performed one-way ANOVAs with false-discovery rate (FDR) correction.

**Table 2:** Purdue Pegboard test scores.

	PD (mean ±1 SD)	PSP (mean ±1 SD)	MSA (mean ±1 SD)	HC (mean ±1 SD)	Diagnosis (*p*-value)	Time (*p*-value)		Time * diagnosis (*p*-value)
**Dominant**								
Visit 1	10.26 ± 2.504	6.590 ± 2.953	6.478 ± 2.761	12.77 ± 2.072	<0.001***	0.660	<0.001***	
Visit 2	9.683 ± 2.581	4.615 ± 2.952	5.304 ± 2.961	13.13 ± 2.631				
Change	−0.579 ± 1.922	−1.974 ± 2.611	−1.174 ± 2.229	0.358 ± 2.497	<0.001***	-	-	
**Non-dominant**								
Visit 1	10.10 ± 2.507	6.179 ± 2.964	6.174 ± 3.128	12.83 ± 1.816	<0.001***	0.833	0.001**	
Visit 2	9.604 ± 2.715	4.410 ± 2.890	5.043 ± 3.282	12.77 ± 1.958				
Change	−0.494 ± 1.901	−1.769 ± 2.757	−1.130 ± 1.984	−0.057 ± 1.748	0.006**	-	-	
**Both hands**								
Visit 1	15.84 ± 4.847	8.718 ± 4.599	9.870 ± 4.770	20.70 ± 3.555	<0.001***	0.793	<0.001***	
Visit 2	14.57 ± 4.791	5.923 ± 4.498	6.957 ± 5.183	21.58 ± 3.845				
Change	−1.268 ± 3.527	−2.795 ± 4.231	−2.913 ± 3.088	0.887 ± 3.625	<0.001***	-	-	
**Assembly**								
Visit 1	18.54 ± 5.377	10.69 ± 5.172	11.48 ± 5.526	26.77 ± 6.381	<0.001***	0.549	0.005**	
Visit 2	18.24 ± 5.887	7.641 ± 4.782	8.696 ± 6.108	26.15 ± 6.320				
Change	−0.305 ± 4.057	−3.051 ± 4.883	−2.783 ± 4.991	−0.623 ± 4.919	0.02*	-	-	

Mean scores (±1 SD) for each Purdue Pegboard task: dominant hand, non-dominant hand, both hands together, and the assembly task for baseline (Visit 1), the 1-year follow-up (Visit 2), and the change (Visit 2 − Visit 1). The main effect of diagnosis, time, and the interaction between time and diagnosis between Visit 1 and Visit 2. Age, site, MOCA baseline score, and sex were included as covariates. Significance indicated by *p < 0.05, **p < 0.01, and ***p < 0.001.

**Fig. 1:**
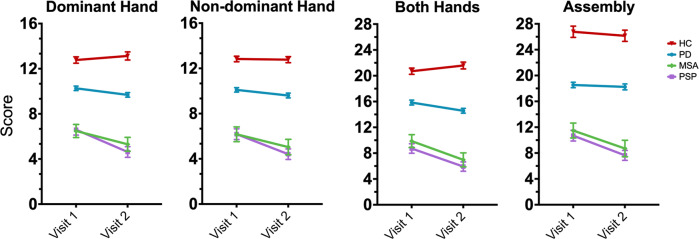
Longitudinal Purdue Pegboard scores. Mean score (±1 SD) on the Purdue Pegboard for Parkinson’s disease (PD), healthy controls (HC), progressive supranuclear palsy (PSP), and multiple system atrophy (MSA) across baseline (Visit 1) and at 1-year follow-up (Visit 2), for all tasks.

The 1-year change for the dominant hand task showed significant declines in PD (*p* = 0.021), MSA (*p* = 0.028), and PSP (*p* < 0.001) groups compared to HCs which had no significant decline (Fig. 1). The PD group showed less decline than PSP (*p* = 0.009), but no significant difference compared to MSA (*p* = 0.393). There was no significant difference in 1-year change between MSA and PSP (*p* = 0.203) for the dominant hand task.

The 1-year change for the PPT non-dominant hand task showed a significant decline in PSP (*p* = 0.004) but no significant difference in the rate of decline in PD (*p* = 0.215) or MSA (*p* = 0.178) compared to HC (Fig. 1). The PD group showed less decline than PSP (*p* = 0.023), but no significant difference compared to MSA (*p* = 0.373). There was no significant difference in 1-year change between MSA and PSP (*p* = 0.308) for the non-dominant hand task.

The 1-year change for the PPT both hands task showed significant declines in PD (*p* = 0.001), MSA (*p* < 0.001), and PSP (*p* < 0.001) groups compared to the HC which had no significant decline (Fig. 1). There was no significant difference in the 1-year change between PD compared to MSA (*p* = 0.060) or PD compared to PSP (*p* = 0.052). There was no significant difference in the 1-year decline in MSA compared to PSP (*p* = 0.862).

The 1-year change for the assembly hands task showed no significant difference between HC compared to PD (*p* = 0.761), MSA (*p* = 0.221), or PSP (*p* = 0.097) (Fig. 1). PD had less decline than PSP (*p* = 0.044), but no significant difference compared to MSA (*p* = 0.097). There was no significant difference in 1-year change between MSA and PSP (*p* = 0.834) for the assembly task.

### Correlations between longitudinal PPT and Parkinsonian motor symptoms

We performed correlations between the 1-year change in MDS-UDPRS-III score with the 1-year change in PPT score, separately for each of the four tasks. Across all disease groups (PD, MSA, and PSP), there was a significant correlation between the 1-year change in MDS-UDPRS-III with the 1-year change in PPT dominant hand (*r*_s_ = −0.234, *p* < 0.001), PPT non-dominant hand (*r*_s_ = −0.169, *p* = 0.011), and PPT both hands (*r*_s_ = −0.151, *p* = 0.024). Subjects that showed worsening PPT scores also had worsening motor symptoms on the MDS-UPDRS-III.

We also looked across each disease group separately. In the PD group, there was a significant correlation between the 1-year change in MDS-UDPRS-III and the 1-year change in the PPT dominant hand (*r*_s_ = −0.179, *p* = 0.021). In the MSA group, there was a significant correlation between the 1-year change in MDS-UDPRS-III with the 1-year change in PPT dominant hand (*r*_s_ = −0.633, *p* = 0.001) and PPT non-dominant hand (*r*_s_ = −0.429, *p* = 0.041). In the PSP group, there were no significant correlations between 1-year change in PPT scores and 1-year change in MDS-UPDRS III scores.

### Predicting PPT from baseline neuroimaging

To determine if PPT scores at 1-year follow-up could be predicted from baseline neuroimaging data, we performed backward linear regression using free water (FW) and free water corrected fractional anisotropy (FAt) values in gray matter regions and white matter tracts relevant to PD and atypical Parkinsonism^4,25–33^. A total of 40 variables were entered in the initial model: age, sex, disease duration, MOCA, and 36 neuroimaging variables. The full list of variables entered into the initial model is described in “Methods”.

In PD subjects, backward linear regression using baseline imaging to predict scores on the PPT both hands task at Visit 2 yielded a significant model with seven predictors [adjusted *R*^2^ = 0.24, F(7,143) = 7.75, *p* < 0.001]. There were five significant predictors in gray matter regions: posterior substantia nigra (pSN) FW (β = −0.33, *p* = 0.013), putamen FW (β = −0.26, *p* = 0.003), pedunculopontine nucleus (PPN) FW (β = 0.53, *p* < 0.001), pSN FAt (β = −0.35, *p* = 0.002), and subthalamic nucleus (STN) FAt (β = 0.41, *p* = 0.001). Sex (β = −0.20, *p* = 0.012) and MOCA score (β = 0.17, *p* = 0.023) were also significant predictors. The final regression model and significant neuroimaging predictors for PD subjects are summarized in Table 3.

**Table 3:** Predicting Purdue Pegboard at follow-up from baseline neuroimaging.

	Standardized Beta Coefficient	T-statistic	*p*-value
**PD [Adjusted** ***R***^**2**^ = **0.24, F(7,143)** = **7.75,** ***p*** < **0.001]**			
*Free-water*			
Putamen	−0.258	−2.993	0.003**
pSN	−0.325	−2.523	0.013*
PPN	0.531	4.393	<0.001***
*Free-water Corrected Fractional Anisotropy*			
pSN	−0.358	−3.168	0.002**
STN	0.406	3.469	0.001**
*Demographics*			
Sex	−0.199	−2.558	0.012*
MOCA	0.172	2.300	0.023*
**Atypical Parkinsonism (MSA and PSP) [Adjusted** ***R***^**2**^ = **0.36, F(7,50)** = **5.52,** ***p*** < **0.001]**			
*Free-water*			
SMA SMATT	−0.389	−2.150	0.036*
STN	−0.439	−2.263	0.028*
Nigrostriatal tract	0.529	2.370	0.022*
*Free-water Corrected Fractional Anisotropy*			
pSN	−0.542	−2.640	0.011*
pre-SMA SMATT	−0.701	−3.188	0.002**
SCP	0.541	2.790	0.007**
Nigrostriatal tract	0.598	3.159	0.003**

Backward linear regression using baseline neuroimaging to predict scores on the PPT both hands task at Visit 2 in individuals with PD and atypical parkinsonism (MSA and PSP). Significance indicated by *p < 0.05, **p < 0.01, and ***p < 0.001.

*pSN* posterior substantia nigra, *PPN* pedunculopontine nucleus, *STN* subthalamic nucleus, *MOCA* Montreal Cognitive Assessment, *pre-SMA* pre-supplementary motor area, *SCP* superior cerebellar peduncle, *SMATT* sensorimotor area tract template.

In subjects with atypical Parkinsonism (MSA and PSP), backward linear regression using baseline imaging to predict scores on the PPT both hands task at Visit 2 yielded a significant model with seven predictors [adjusted R^2^ = 0.36, F(7,50) = 5.52, *p* < 0.001]. Two predictors were from gray matter: pSN FAt (β = −0.54, *p* = 0.011) and STN FW (β = −0.43, *p* = 0.028). Five predictors were from white matter tracts: supplementary motor area white matter tract (SMA SMATT) FW (β = −0.389, *p* = 0.036), pre-supplementary motor area white matter tract (preSMA SMATT) FAt (β = −0.70, *p* = 0.002), superior cerebellar peduncle (SCP) FAt (β = 0.54, *p* = 0.007), nigrostriatal tract FAt (β = 0.60, *p* = 0.003), and nigrostriatal tract FW (β = 0.53, *p* = 0.022). The final regression model and significant neuroimaging predictors for subjects with atypical Parkinsonism (MSA and PSP) are summarized in Table 3.

### PPT accelerometry—demographic and clinical data

There was no significant difference in age [t(37) = 0.517, *p* = 0.608] and MOCA score [t(32) = 0.517, *p* = 0.694] between HCs and PD. Five HC participants did not have a MOCA score and were excluded from this *t*-test. There was a significant difference in MDS-UPDRS-III score [t(37) = 12.17, *p* < 0.001] between HCs and PD, which was expected. The demographic and clinical data for the accelerometer cohort are summarized in Table 4.

**Table 4:** Participant demographic information—Purdue Pegboard accelerometry.

Demographic	PD (14M/ 6F)	HC (13M/ 6F)	*p*-value
Age	65.00 ± 8.072	63.47 ± 10.23	0.608
Disease duration (years)	2.958 ± 2.172	-	-
MOCA	26.75 ± 2.712	27.07 ± 1.592	0.347
MDS-UPDRS-III	39.20 ± 12.04	4.110 ± 3.665	<0.001***

Participant sex, age, MOCA score, and disease duration (mean ± 1 SD) for Purdue Pegboard accelerometry. The Montreal Cognitive Assessment (MOCA) and Movement Disorders Society Unified Parkinson’s Disease Rating Scale Part III (MDS-UPDRS-III) include mean ± 1 SD. T-tests were completed to see if groups were similar or different from each other. Significance indicated by *p < 0.05, **p < 0.01, and ***p < 0.001.

### PPT accelerometry—standard deviation of acceleration

**Fig. 2:**
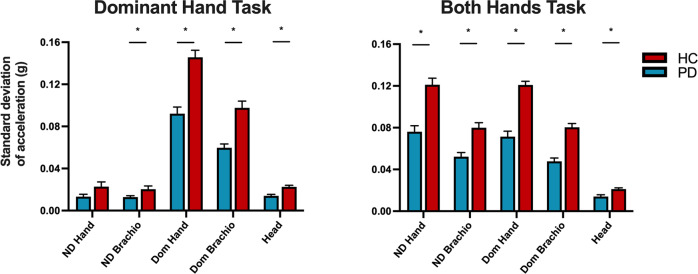
Standard deviation of acceleration during Purdue Pegboard test. Group means for the standard deviation of acceleration (±1 SEM) during the dominant hand task (left) and both hands tasks (right) of the Purdue Pegboard in Parkinson’s disease (PD) and healthy controls (HC). Significant group differences are indicated with an asterisk *p < 0.05, FDR-corrected.

Group means and statistics comparing the standard deviation of acceleration between PD and HC participants are summarized in Table 5. For the dominant hand task, we observed a significant main effect of diagnosis [F(1,35) = 32.15, *p* < 0.001] and sensor [F(4,140) = 10.27, *p* < 0.001]. There was also a significant interaction between diagnosis and sensors [F(4,140) = 18.68, *p* = 0.004]. Post hoc comparisons for the dominant hand task revealed that PD subjects had significantly lower standard deviations of acceleration (*p*_FDR _< 0.05) for sensors non-dominant brachioradialis (NDB), dominant hand (DH), dominant brachioradialis (DB), and head (HD) (Fig. 2, Table 5).

**Table 5:** Purdue Pegboard accelerometry—standard deviation of acceleration.

Task	Parkinson’s disease (*n* = 20)		Healthy control (*n* = 19)	T-statistic	*p*-value	*p*-value (FDR-corrected)
**Dominant**						
NDH		0.013 ± 0.010	0.023 ± 0.019	1.944	0.060	0.060
NDB		0.013 ± 0.005	0.020 ± 0.014	2.269	0.029*	0.037*
DH		0.092 ± 0.028	0.146 ± 0.029	5.852	<0.001***	<0.001***
DB		0.060 ± 0.016	0.098 ± 0.028	5.200	<0.001***	<0.001***
HD		0.014 ± 0.006	0.023 ± 0.006	4.113	<0.001***	<0.001***
**Non-dominant**						
NDH		0.100 ± 0.033	0.149 ± 0.032	4.753	<0.001***	<0.001***
NDB		0.067 ± 0.023	0.096 ± 0.025	3.790	<0.001***	<0.001***
DH		0.016 ± 0.019	0.021 ± 0.017	0.880	0.385	0.385
DB		0.015 ± 0.012	0.019 ± 0.010	1.014	0.317	0.385
HD		0.015 ± 0.006	0.020 ± 0.006	2.522	0.016*	0.027*
**Both**						
NDH		0.076 ± 0.026	0.121 ± 0.027	5.268	<0.001***	<0.001***
NDB		0.052 ± 0.018	0.080 ± 0.021	4.461	<0.001***	<0.001***
DH		0.071 ± 0.023	0.121 ± 0.016	7.731	<0.001***	<0.001***
DB		0.048 ± 0.019	0.080 ± 0.016	6.805	<0.001***	<0.001***
HD		0.014 ± 0.007	0.021 ± 0.005	3.565	0.001**	0.001**
**Assembly**						
NDH		0.058 ± 0.019	0.096 ± 0.031	4.618	<0.001***	<0.001***
NDB		0.043 ± 0.013	0.064 ± 0.023	3.610	0.001**	0.001**
DH		0.056 ± 0.016	0.089 ± 0.023	5.116	<0.001***	<0.001***
DB		0.040 ± 0.011	0.061 ± 0.018	4.352	<0.001***	0.001**
HD		0.012 ± 0.003	0.017 ± 0.004	3.775	0.001**	0.001**

Group means for the standard deviation of acceleration (±1 SD) following 1–50 Hz bandpass filtering for a subset of participants with Parkinson’s disease (PD) and healthy controls (HC). Group comparisons between each sensor were calculated with false-discovery rate correction (FDR). Significance indicated by *p < 0.05, **p < 0.01, and ***p < 0.001.

*NDH* non-dominant hand, *NDB* non-dominant brachioradialis, *DH* dominant hand, *DB* dominant brachioradialis, *HD* head.

For the non-dominant hand task, we observed a significant main effect of diagnosis [F(1,35) = 17.01, *p* < 0.001] and sensor [F(4,140) = 5.16, *p* < 0.001]. There was also a significant interaction between diagnosis and sensors [F(4,140) = 13.54, *p* < 0.001]. Post hoc comparisons for the non-dominant hand task revealed that PD patients had a significantly lower standard deviation of acceleration (*p*_FDR _< 0.05) for sensors non-dominant hand (NDH), NDB, and HD (Fig. 2, Table 5).

For the both hands task, we observed a significant main effect of diagnosis [F(1,35) = 38.94, *p* < 0.001] and sensor [F(4,140) = 5.99, *p* < 0.001]. There was also a significant interaction between diagnosis and sensors [F(4,140) = 20.02, *p* < 0.001]. Post hoc comparisons for the both hands task revealed that PD patients had a significantly lower standard deviation of acceleration (*p*_FDR _< 0.05) for all sensors (Fig. 2, Table 5).

For the assembly task, we observed a significant main effect of diagnosis [F(1,35) = 30.74, *p* < 0.001] and sensor [F(4,140) = 5.10, *p* < 0.001]. There was also a significant interaction between diagnosis and sensors [F(4,140) = 8.82, *p* < 0.001]. Post hoc comparisons for the assembly task revealed that PD patients had a significantly lower standard deviation of acceleration (*p*_FDR _< 0.05) for all sensors (Fig. 2, Table 5).

### PPT accelerometry—approximate entropy

**Fig. 3:**
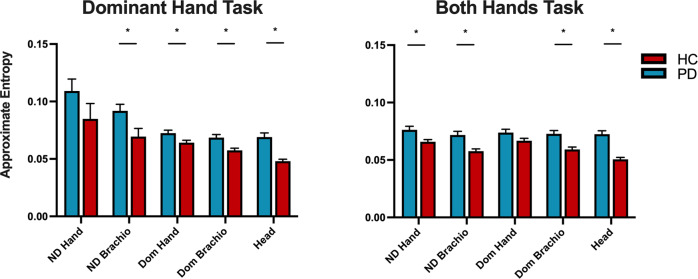
Approximate entropy during Purdue Pegboard test. Group means for approximate entropy of acceleration (±1 SEM) during the dominant hand task (left) and both hands tasks (right) of the Purdue Pegboard in Parkinson’s disease (PD) and healthy controls (HC). Significant group differences are indicated with an asterisk *p < 0.05, FDR-corrected.

Approximate entropy characterizes the regularity and predictability of sequential changes in time-series data^34^. Group means and statistics comparing approximate entropy between PD and HC participants are summarized in Table 6. For the dominant hand task, we observed a significant main effect of the group [F(1,35) = 8.96, *p* = 0.005]. There was no main effect of the sensor and no significant interaction between the group and the sensor. Post hoc comparisons for the dominant hand task revealed that PD patients had significantly higher approximate entropy (*p*_FDR _< 0.05) for sensors of the NDB, DH, DB, and HD (Fig. 3, Table 6).

**Table 6:** Purdue Pegboard accelerometry—approximate entropy.

Task	Parkinson’s disease (*n* = 20)		Healthy Control (*n* = 19)	T-statistic	*p*-value	*p*-value (FDR-corrected)
**Dominant**						
NDH		0.109 ± 0.046	0.085 ± 0.058	1.447	0.156	0.156
NDB		0.092 ± 0.026	0.069± 0.031	2.472	0.018*	0.023*
DH		0.072 ± 0.011	0.064 ± 0.009	2.494	0.017*	0.023*
DB		0.069 ± 0.012	0.057 ± 0.008	3.267	0.002**	0.006**
HD		0.069 ± 0.016	0.048 ± 0.007	5.255	<0.001***	0.001***
**Non-dominant**						
NDH		0.072 ± 0.013	0.065 ± 0.008	1.936	0.061	0.101
NDB		0.067 ± 0.013	0.057 ± 0.009	2.862	0.007*	0.017*
DH		0.112 ± 0.057	0.092 ± 0.059	1.102	0.278	0.278
DB		0.090 ± 0.030	0.076 ± 0.030	1.371	0.179	0.223
HD		0.065 ± 0.016	0.049 ± 0.009	3.808	<0.001***	0.003**
**Both**						
NDH		0.076 ± 0.014	0.066 ± 0.008	2.793	0.008**	0.010*
NDB		0.072 ± 0.014	0.058 ± 0.009	3.720	<0.001***	0.001**
DH		0.074 ± 0.013	0.067 ± 0.009	1.990	0.054	0.054
DB		0.072 ± 0.013	0.059 ± 0.009	3.759	<0.001***	0.001*
HD		0.072 ± 0.013	0.051 ± 0.008	6.232	<0.001***	<0.001***
**Assembly**						
NDH		0.066 ± 0.012	0.060 ± 0.013	1.524	0.136	0.136
NDB		0.067 ± 0.012	0.059 ± 0.011	2.170	0.037*	0.056
DH		0.070 ± 0.011	0.063 ± 0.010	2.077	0.045*	0.056
DB		0.070± 0.013	0.059 ± 0.010	2.888	0.006*	0.016*
HD		0.077 ± 0.013	0.058 ± 0.007	5.502	<0.001***	<0.001***

Group means for approximate entropy (±1 SD) following 1–50 Hz bandpass filtering for a subset of participants with Parkinson’s disease (PD, *n* = 20) and healthy controls (HC, *n* = 19). Post hoc comparisons were calculated with false-discovery rate (FDR) correction. Significance indicated by *p < 0.05, **p < 0.01, and ***p < 0.001.

*NDH* non-dominant hand, *NDB* non-dominant brachioradialis, *DH* dominant hand, *DB* dominant brachioradialis, *HD* head.

For the non-dominant hand task, we observed a significant main effect of the group [F(1,35) = 4.129, *p* = 0.05]. There was no main effect of the sensor and no significant interaction between the group and the sensor. Post hoc comparisons for the non-dominant hand task revealed that PD patients had significantly higher approximate entropy (*p*_FDR _< 0.05) for sensors of the NDB and HD (Fig. 3, Table 6).

For the both hands task, we observed a significant main effect of the group [F(1,35) = 19.66, *p* < 0.001] and sensor [F(4,140) = 2.70, *p* = 0.033]. There was also a significant interaction between the group and the sensor [F(4,140) = 6.52, *p* < 0.001]. Post hoc comparisons for the both hands task revealed that PD patients had significantly higher approximate entropy (*p*_FDR _< 0.05) for sensors NDH, NDB, DB, and HD (Fig. 3, Table 6).

For the assembly task, we observed a significant main effect of the group [F(1,35) = 11.481, *p* = 0.002]. There was no main effect of the sensor. There was a significant interaction between the group and the sensor [F(4,140) = 5.47, *p* < 0.001]. Post hoc comparisons for the assembly task revealed that PD patients had significantly higher approximate entropy (*p*_FDR _< 0.05) for sensors DB and HD (Fig. 3, Table 6).

### Correlations between PPT score and accelerometry

We also investigated the relationship between PPT scores and accelerometry measures on a task-by-task basis. In PD patients, we found that there was a significant correlation between PPT score and accelerometer measures for the dominant hand task, non-dominant hand task, and both hands task. Dominant hand PPT score was significantly correlated with DH standard deviation of acceleration (*r* = 0.47, *p* = 0.036), HD standard deviation of acceleration (*r* = 0.527, *p* = 0.017), and HD approximate entropy (*r* = −0.472, *p* = 0.036). Non-dominant hand PPT score was significantly correlated with NDH standard deviation of acceleration (*r* = 0.485, *p* = .030) and NDB approximate entropy (*r* = −0.448, *p* = 0.048). For the both hands task, the PPT score was significantly correlated with DH standard deviation of acceleration (*r* = 0.686, *p* < 0.001), NDH standard deviation of acceleration (*r* = 0.643, *p* = 0.002), and HD approximate entropy (*r* = −0.574, *p* = 0.008).

## Discussion

Objective measures of motor decline in PD and other forms of Parkinsonism are critically needed for tracking disease progression and evaluating treatment outcomes. The PPT is objective, reliable, and inexpensive to administer. Moreover, the PPT has been shown to be sensitive to progression effects in PD^19,20^. In this study, we assessed longitudinal changes in PPT performance in a large multisite cohort of patients with PD, MSA, PSP, and similarly aged HCs. We show four key findings from this study. (1) Parkinsonian patients had a significant decline in PPT performance after 1 year, whereas HCs did not. Specifically, Parkinsonian patients showed a longitudinal decline for the dominant hand and both hands PPT tasks. (2) Parkinsonian patients showed a significant correlation between the decline in PPT score and the progression of motor symptoms, measured by the MDS-UPDRS-III. (3) PPT performance at longitudinal follow-up was predicted by the patient’s baseline free-water imaging measures from the basal ganglia for PD, and from the basal ganglia, cerebellum, and cortex for atypical Parkinsonism. (4) Accelerometry in a subset of patients with PD revealed greater variability and irregularity of upper limb acceleration, and these measures correlated with PPT scores, suggesting that these kinematic factors contribute to PD patients’ poor performance on the PPT.

Other work has demonstrated that the PPT is sensitive for detecting longitudinal changes in large cohorts of subjects with PD^19,20^, but those studies did not include a healthy aging control group. In this study, we replicated and expanded upon those findings in a larger multisite study, demonstrating that PPT performance decline in Parkinsonism is a reliably observed phenomenon. Other measures of motor function have been proposed for the assessment of progression effects in PD^21^. Although these assessments share many of the appealing qualities of the PPT, such as ease of administration and high test-retest reliability, standard implementation of these tasks is not sensitive to longitudinal progression effects in PD^22,23^. An alternative conceptual approach for quantifying progression effects in PD has been suggested, whereby smartphones can be used to gather large amounts of data, and machine learning approaches can be used to sift through those data for meaningful signatures of disease severity and progression. Zhan et al.^35^ performed a study where smartphones were utilized to gather 6148 activity assessments from 129 subjects, and machine learning approaches were used to derive a composite score of disease severity. Although conceptually appealing, such an approach may be difficult to implement on the scale of a clinical trial or large multisite study due to factors such as the cost of smartphones, continual software upgrades, and battery-life issues that can affect the devices.

In the longitudinal assessment of PPT scores, we observed a significant interaction between Parkinsonian diagnoses and time on all four PPT tasks. HCs showed no longitudinal decline in PPT performance, whereas patients with Parkinsonism showed a significant decline in PPT scores over time. Subjects with atypical Parkinsonism (MSA and PSP) had worse scores compared to HC and PD at both time points, but there was no difference between MSA and PSP at either time point. The dominant hand and both hands PPT tasks were most sensitive to disease progression (Fig. 1). In addition, we show that worsening PPT scores in patients with Parkinsonism were significantly correlated with the progression of motor symptoms on the MDS-UPDRS-III. It is worth noting that subjects in the PSP group were slightly older than those in PD, MSA, and HC groups. We also observed significantly lower MOCA scores in MSA and PSP patients, indicating mild cognitive impairment, which is not uncommon in individuals with these more quickly progressing forms of atypical Parkinsonism. We statistically controlled for these differences on all analyses of PPT scores by covarying the factors of testing site, age, disease duration, and MOCA score. These findings bolster the notion that PPT is sensitive for detecting longitudinal changes in PD and atypical Parkinsonism, even when accounting for factors such as age, disease duration, and cognitive impairment.

We further demonstrate that changes in PPT score are associated with underlying neuropathology in Parkinsonism by showing that PPT performance at longitudinal follow-up was able to be predicted by the patient’s baseline free-water imaging measures. Free-water imaging is a sensitive measure of neurodegeneration and neuroinflammation in Parkinsonism, and we selected these regions based on their relevance to Parkinsonism from findings in cross-sectional and longitudinal studies^4,25–33^. The both hands task was chosen as the outcome variable for the regression analysis because it was the most sensitive of the four PPT tasks to longitudinal progression effects in Parkinsonism. In PD patients, we found that subcortical free water imaging measures, sex, and MOCA score were significant predictors of PPT score (Table 3). The other statistical comparisons employed in this manuscript covaried out the effects of sex and MOCA, whereas backward linear regression methods evaluate these variables in the presence of other predictors. This finding that it is a significant predictor of PPT score is likely because males with PD have been shown to have a greater rate of disease progression and greater risk of developing cognitive impairment than females with PD^36^. In patients with atypical Parkinsonism, we found that both cortical and subcortical free water imaging measures were significant predictors of PPT score (Table 3). This finding matches our prior work, which showed that free water imaging revealed extra-nigral and extra-striatal longitudinal progression effects in atypical Parkinsonian disorders, whereas those in PD were primarily localized to basal ganglia and midbrain^37^. Interestingly, the involvement of the PPN in PD patients reflects growing evidence for its involvement in motor control in PD^38^. The involvement of SCP in predicting PPT score at follow-up in atypical Parkinsonism is also reassuring, as degeneration of this tract is a hallmark of disease progression in PSP^39^.

Performance on the PPT or similar pegboard tasks has also been linked to other indicators of disease pathology and treatment effects in PD. Lower F-DOPA PET uptake in the striatum was strongly correlated with lower PPT scores, indicating that more severe nigrostriatal degeneration was associated with worse Pegboard scores in PD patients^40^. Scores on a modified pegboard task were sensitive to the levodopa challenge, where scores improved at 30, 60, and 90 min after levodopa administration and were correlated to changes in MDS-UPDRS-III scores over the same time course^41^. A study of PD patients who received bilateral deep brain stimulation of the subthalamic nucleus showed that PPT scores improved post-operatively for the dominant hand and both hands tasks^42^, the same tasks that we observed longitudinal changes across all three Parkinsonian populations in this study.

We next sought to characterize kinematic features that contribute to poor performance on the PPT in PD patients. In a subset of subjects, we performed triaxial accelerometry to quantify the variability and irregularity of upper limb acceleration. Since PD patients move more slowly, we anticipated that the standard deviation of acceleration could be reduced in PD compared with controls. We confirmed this hypothesis by finding a lower standard deviation of acceleration during all tasks. The sensors that showed the greatest differences between PD and healthy controls were those specific to the task being performed (e.g., DH and DB during the dominant hand task). In other paradigms, it has been observed that patients with PD have a greater degree of speed-accuracy trade-off than healthy controls^24^. Since the PPT task requires a high degree of accuracy when placing the pegs in the holes, one interpretation of the data is that PD patients were slowing down in order to complete the task.

Several studies have also examined the temporal organization of motor output in PD using approximate entropy. Resting and postural tremor in PD were shown to have lower approximate entropy, indicating a more regular temporal organization of tremor^43^. Advanced-stage PD patients were shown to have increased approximate entropy during a spiral drawing task^44^, demonstrating more temporal irregularity during goal-oriented motor behavior. In the present study, subjects performed the PPT, which is a repetitive goal-oriented task that should have a low approximate entropy if performed perfectly due to the rhythmic nature of the task^45^. We observed that approximate entropy was higher in PD patients than controls during the PPT, which we interpret as a disruption of the appropriate temporal organization for this motor output. Another study developed an algorithm to quantify motor states in PD based on accelerometry during an alternating hand pronation-supination test, and two of the three most important features in the final model were approximate entropy and standard deviation of acceleration^46^. Together these studies indicate that the metrics of the standard deviation of acceleration and approximate entropy are highly relevant kinematic features of upper limb disturbances in PD and can be extended to help explain the deficits shown by PD patients on the PPT.

When evaluating factors for clinical trials or large multisite studies of disease progression in PD, the cost is a major consideration. Consider a hypothetical multisite trial with 300 subjects, 50 sites, and 3 time points. Let us assume that DAT SPECT evaluation costs approximately $2500 per scan, whereas a PPT costs $150 per device and can be used at each time point for all subjects at a given site. The estimated cost of DAT SPECT scans would be $2,250,000 ($2500 × 300 subjects × 3 time points), whereas the cost of PPT would only be $7500 ($150 × 50 sites). This hypothetical estimate demonstrates the cost-effectiveness of PPT in a clinical trial.

In this study, we demonstrate that patients with PD and atypical Parkinsonism show a predictable 1-year decline in the PPT that is not present in healthy aging controls. We also show that these declines in PPT scores were related to the progression of motor symptom severity. All patients in this study were tested after overnight withdrawal from dopaminergic medications in order to focus on disease progression and minimize the impact of medication-related changes over 1 year. We further link PPT performance to free-water imaging metrics, which are sensitive to pathological brain changes in Parkinsonism. Lastly, we used accelerometry to quantify performance deficits on the PPT in PD subjects compared to controls, showing that PD patients had greater variability and irregularity of upper limb acceleration during these tasks, which significantly correlated with PPT scores on those tasks. These kinematic features contributing to fine motor deficits in the PPT in PD have also been shown in other quantitative studies of motor dysfunction PD. It is important to note that the PPT is limited to the upper extremities and that other measures of lower extremity function and mobility may be needed for assessing the progression of PD. This study, in conjunction with other discussed literature, suggests that PPT performance is a useful and objective marker of disease progression and treatment effects in Parkinsonism that parallels measures of disease pathology. Thus, the PPT may provide utility for studying disease progression in PD and atypical Parkinsonism as it is inexpensive and easy to administer while providing relevant and objective readouts of fine motor dysfunction.

## Methods

### Subjects

The study included 164 subjects with PD, 39 with PSP, 23 with MSA, and 53 HC, all between 38 and 81 years of age. Subjects were referred from and diagnosed by movement disorder specialists at the University of Florida (UF) and Northwestern University (NU). All procedures were approved by the Institutional Review Board at each site, and written informed consent was obtained from all subjects in accordance with the Declaration of Helsinki. Subjects in the PD group included 107 males and 57 females. Subjects in the PSP group included 17 males and 22 females. Subjects in the MSA group included 17 males and 6 females. Subjects in the HC group included 20 males and 33 females. Participant demographics are shown in Table 1.

### Experimental design

Subjects were tested at baseline (Visit 1) and a 1-year follow-up (Visit 2). This was carried out at UF or NU. Subjects taking dopaminergic medication went through overnight withdrawal (>12 h) of the medication. Overnight withdrawal was done to focus on disease progression and minimize the impact of medication changes over 1 year. Motor impairment was assessed using MDS-UPDRS-III. Cognitive function was assessed using the Montreal Cognitive Assessment (MOCA)^47^. Subjects were given clinical assessments, performed the PPT, and underwent neuroimaging at both Visit 1 and Visit 2.

The PPT included four tasks: dominant hand, non-dominant hand, both hands simultaneously, and assembly task. Subjects were instructed to pick up a small metal peg out of a storage area at the top of the board and place it into a series of vertically aligned holes on the board and to do this for as many pegs as possible over 30 s. This objective is carried out in three of the four tasks for the dominant hand, non-dominant hand, and simultaneously using both hands. The fourth task is an assembly task and is performed over 60 s, where subjects assemble multiple components into a unit, which is made by placing a peg in a hole (dominant hand), placing a washer over the peg (non-dominant hand), then a small cylindrical collar (dominant hand), followed by a second washer on top (non-dominant hand).

To determine the kinematic motor deficits that underlie changes in Purdue Pegboard performance in a subset of subjects (20 PD and 19 HC, all taken from UF), the PPT was monitored during one of their visits with a series of triaxial accelerometers, placed on the following areas: non-dominant hand (NDH), non-dominant brachioradialis (NDB), dominant hand (DH), dominant brachioradialis (DB), and forehead (HD) (Supplementary Fig. 1). Three PD subjects and two HC subjects were left hand dominant, and accelerometer data were analyzed accordingly. Accelerometer data were collected with the Delsys Trigno Wireless System (Delsys Inc., Boston, MA) using MotionMonitor software (Innovative Sports Training, Inc., Chicago, IL) at a sampling rate of 2000 Hz. Acceleration data from a representative PD and HC participant performing the PPT with their dominant (right) hand are depicted in Supplementary Fig. 2. Data processing was performed using a custom script in MATLAB (2020b, The MathWorks, Inc.). We extracted the acceleration time series for each plane of motion (*x*, *y*, *z*) for each of the five sensors during each PPT task. Each time series went through spike removal and bandpass filtering between 1 and 50 Hz (Butterworth 4th order dual pass), followed by Fourier transform^48–50^. In order to characterize fluctuations and regularity of upper limb acceleration during these tasks, we measured the standard deviation of acceleration and approximate entropy of each sensor^34^. These measures were selected because they have been shown to be important features discriminating individuals with PD from healthy controls^44–46,51^. We averaged these values across the *x*, *y*, and *z* planes to generate a single measure for each sensor, as was done in previous work^52^.

### Neuroimaging acquisition and processing

Data were acquired at the UF McKnight Brain Institute (3T Siemens Prisma) and NU Center for Translational Imaging (3T Siemens Prisma Fit) using a 64-channel head coil. Identical scan parameters were used to acquire data from both sites, and previously published quality assurance data indicate signal integrity remained stable over time at both sites^53^. T1-weighted images (repetition time: 2000 ms, echo time: 2.99 ms, flip angle: 8°, TI = 1010 ms, GRAPPA factor = 2, 0.8 mm isotropic voxels, bandwidth: 240 Hz/pixel) were acquired with a three-dimensional (3D) magnetization-prepared 180° radio-frequency pulses and rapid gradient-echo (MP-RAGE) sequence in 208 contiguous sagittal slices. Single-shell diffusion MRI images (repetition time: 6400 ms, echo time: 58 ms, flip angle: 90°, field of view: 256 × 256 mm, resolution: 2 mm isotropic, 64 diffusion gradient directions, b-values: 5 × 0, and 64 × 1000 s/mm^2^, 69 axial slices, bandwidth: 2442 Hz/pixel, total acquisition time: 7 min 41 s) were acquired for free-water imaging.

Diffusion MRI data processing was performed, consistent with previously published studies^37,53^. Free-water (FW) and free-water corrected fractional anisotropy (FAt) images were generated using custom-written MATLAB (2020b, The MathWorks, Inc.) code, as described in previous work^26,30,53–56^. These FW and FAt images were non-linearly transformed into MNI space using Advanced Normalization Tools (ANTs)^57^ and in-house templates^54^.

### Statistical analysis

All test statistics were performed as two-tailed, where applicable. To evaluate cross-sectional group differences between PD, PSP, MSA, and HC subjects on age and disease duration, we performed one-way ANOVAs for Visit 1 only. For MOCA and MDS-UPDRS-III scores, we performed one-way ANOVAs, separately for Visit 1 and Visit 2. To evaluate progression effects on the PPT, we performed two-factor (2×4) repeated measures ANOVAs for each of the four tasks (dominant, non-dominant, both, assembly). Time (Visit 1, Visit 2) was a within-subjects factor, and diagnosis (PD, MSA, PSP, or HC) was a between-subjects factor. Age, testing site, Visit 1 MOCA score, and sex were included as covariates. In order to evaluate the change from baseline to 1-year follow-up on a subject-by-subject basis, we calculated the 1-year change in score (PPT_visit2_ − PPT_visit1_) for each task and compared it between groups using one-way ANOVA. False-discovery rate (FDR) correction for multiple comparisons was used for post hoc tests. We also performed Spearman’s rank-order correlations between the 1-year change in MDS-UDPRS-III score (non-normal distribution) with the 1-year change in PPT score (normal distribution) separately for each of the four tasks.

To determine if the PPT score at 1-year follow-up could be predicted from baseline neuroimaging data, we performed backward linear regression using FW and FAt values in gray matter regions and white matter tracts relevant to PD and atypical Parkinsonism. We chose scores on the PPT both hands task as the dependent variable to be predicted because free-water imaging data were derived from regions of interest across both hemispheres of the brain. A total of 40 variables were entered in the initial model: age, sex, disease duration, MOCA, and 36 imaging variables. At each step, the least significant predictor was removed from the regression model until only those with *p* < 0.05 remained. For imaging predictors, there were 18 regions or tracts of interest, and the FW and FAt values for each were included. Thirteen gray matter regions were included from the Parkinson’s disease region of interest template validated in previous work by our group^55^. These were primary motor cortex (M1), supplementary motor area (SMA), pre-supplementary motor area (preSMA), superior frontal gyrus (SFG), middle frontal gyrus (MFG), inferior parietal lobule (IPL), supramarginal gyrus (SMG), caudate, putamen, subthalamic nucleus (STN), posterior substantia nigra (pSN), pedunculopontine nucleus (PPN), and dentate nucleus of the cerebellum. A total of five white matter tracts were investigated. Three white matter tracts were from the sensorimotor area tract template (SMATT)^58^, which included descending sensorimotor tracts from M1, SMA, and preSMA. One tract from the cerebellar probabilistic white matter atlas^59^, the superior cerebellar peduncle (SCP). One tract was from the Parkinson’s disease region of interest template^55^, the nigrostriatal tract. We also included baseline MOCA scores to account for possible effects of cognitive function, as well as age and sex. We performed two separate regressions, one for only PD subjects and one for atypical Parkinsonism (MSA and PSP).

For accelerometer data, *t*-tests were used to see if there were any differences between HCs and PD participants for age, MOCA score, and MDS-UPDRS-III. Disease duration (in years), mean, and standard deviation was also reported for PD participants. The standard deviation of acceleration and approximate entropy were separately evaluated using two-factor (2×5) repeated measures ANOVAs for each of the four tasks (dominant hand, non-dominant hand, both hands, assembly). Sensor (NDH, NDB, DH, DB, HD) was a within-subjects factor, and diagnosis (PD or HC) was a between-subjects factor. Age, MOCA score, and sex were included as covariates. For the post hoc analysis, we performed one-way ANOVAs within each task to identify which sensors had statistical significance, with *p*-values corrected for multiple comparisons across sensors using the false-discovery rate (FDR) method^60^. We also performed correlations between accelerometer measures and PPT score were performed using Pearson’s correlation coefficient, as these measures were both normally distributed.

### Reporting summary

Further information on research design is available in the Nature Research Reporting Summary linked to this article.

## Supplementary information

Supplementary Information

Reporting Summary

## Data Availability

The clinical and neuroimaging data in this study are openly available in the Parkinson’s disease biomarker program (https://pdbp.ninds.nih.gov). Accelerometer data can be requested by contacting the corresponding author.

## Code availability

Accelerometer data processing was performed using a custom workflow in MATLAB. The steps of the workflow are described in “Methods” and can be requested by contacting the corresponding author.
