# Supplementary material for: Distinct cortical and subcortical predictors of Purdue Pegboard decline in Parkinson’s disease and atypical parkinsonism
Source: NPJ Parkinsons Dis. 2023 Jun 5;9:85. doi: 10.1038/s41531-023-00521-0 (PMC10241903; doi:10.1038/s41531-023-00521-0)
Supplement: Supplementary file 1 — Supplementary Information [file 41531_2023_521_MOESM1_ESM.pdf]

Abbreviations

ANOVA	=	Analysis of Variance
DAT SPECT	=	Dopamine Transporter – Single Photon Emission Computer Tomography
DB	=	Dominant Brachioradialis
DH	=	Dominant Hand
F-DOPA PET	=	Flourodopa F 18 Positron Emission Tomography
HC	=	Healthy Controls
HD	=	Head
MDS-UPDRS	=	Movement Disorder Society – Unified Parkinson's Disease Rating Scale
MDS-UPDRS-III	=	Movement Disorder Society – Unified Parkinson's Disease Rating Scale Part III
MOCA	=	Montreal Cognitive Assessment
MSA	=	Multiple System Atrophy
NDB	=	Non-Dominant Brachioradialis
NDH	=	Non-Dominant Hand
NU	=	Northwestern University
PD	=	Parkinson's Disease
PPT	=	Purdue Pegboard Test
PSP	=	Progressive Supranuclear Palsy
UF	=	University of Florida

Neuroimaging Abbreviations

FAt	=	Free Water Corrected Fractional Anisotropy
FW	=	Free Water
IPL	=	Inferior Parietal Lobe
M1	=	Primary Motor Cortex
MFG	=	Middle Frontal Gyrus
PPN	=	Pedunculopontine Nucleus
preSMA	=	Pre-supplementary Motor Area
pSN	=	Posterior Substantia Nigra
SCP	=	Superior Cerebellar Peduncle
SFG	=	Superior Frontal Gyrus
SMA	=	Supplementary Motor Area
SMATT	=	Sensorimotor Area Tract Template
SMG	=	Supramarginal Gyrus
STN	=	Subthalamic Nucleus

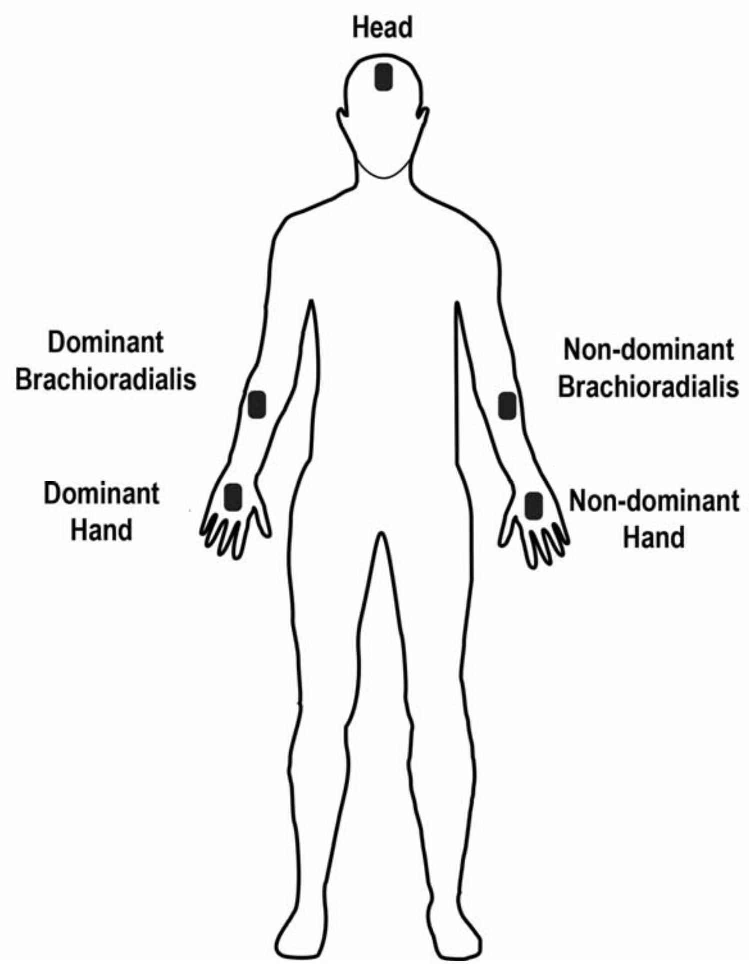

Supplementary Figure 1. Accelerometer placement. Depiction of accelerometer placement for a right hand dominant individual for Purdue Pegboard accelerometry, performed in a subset of healthy controls (HC) and Parkinson's disease (PD) patients.

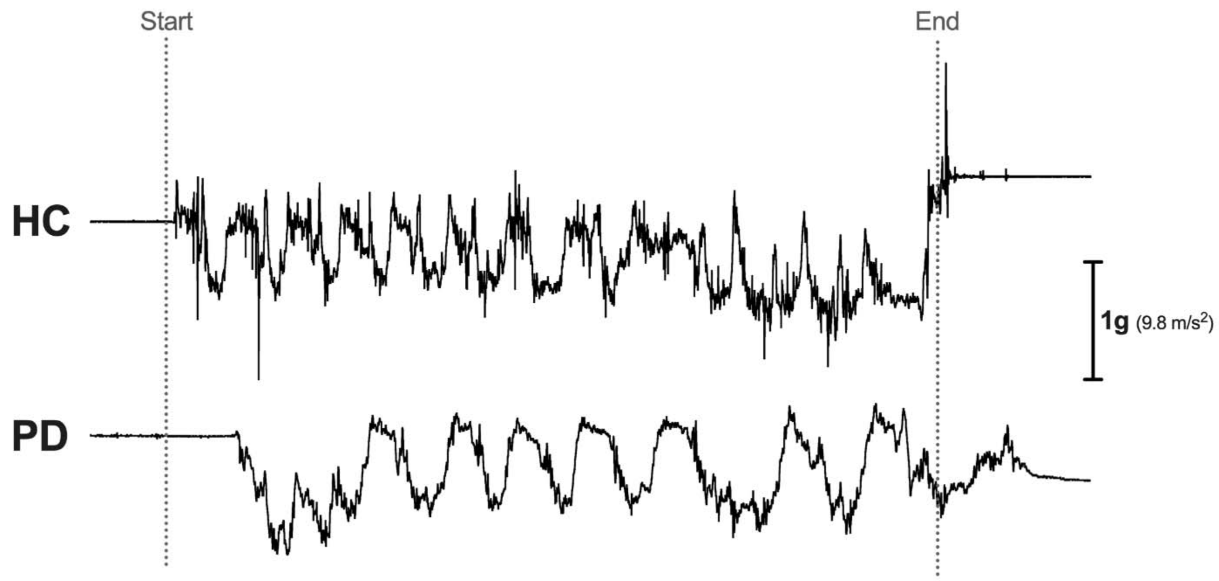

Supplementary Figure 2. Accelerometer signal during Purdue Pegboard test. Raw acceleration signal of the dominant hand (DH) during the Purdue Pegboard dominant hand task from a representative healthy control (HC) and Parkinson's disease (PD) participant.
