# Supplementary material for: Distinct cortical and subcortical predictors of Purdue Pegboard decline in Parkinson’s disease and atypical parkinsonism
Source: NPJ Parkinsons Dis. 2023 Jun 5;9:85. doi: 10.1038/s41531-023-00521-0 (PMC10241903; doi:10.1038/s41531-023-00521-0)
Supplement: Supplementary file 2 — Reporting Summary [file 41531_2023_521_MOESM2_ESM.pdf]

nature portfolio

Corresponding author(s): Bradley J. Wilkes

Last updated by author(s): Apr 10, 2023

Reporting Summary

Nature Portfolio wishes to improve the reproducibility of the work that we publish. This form provides structure for consistency and transparency in reporting. For further information on Nature Portfolio policies, see our Editorial Policies and the Editorial Policy Checklist.

Statistics

For all statistical analyses, confirm that the following items are present in the figure legend, table legend, main text, or Methods section.

n/a	Confirmed	
☐	☒	The exact sample size (*n*) for each experimental group/condition, given as a discrete number and unit of measurement
☐	☒	A statement on whether measurements were taken from distinct samples or whether the same sample was measured repeatedly
☐	☒	The statistical test(s) used AND whether they are one- or two-sided<br>*Only common tests should be described solely by name; describe more complex techniques in the Methods section.*
☐	☒	A description of all covariates tested
☐	☒	A description of any assumptions or corrections, such as tests of normality and adjustment for multiple comparisons
☐	☒	A full description of the statistical parameters including central tendency (e.g. means) or other basic estimates (e.g. regression coefficient) AND variation (e.g. standard deviation) or associated estimates of uncertainty (e.g. confidence intervals)
☐	☒	For null hypothesis testing, the test statistic (e.g. *F*, *t*, *r*) with confidence intervals, effect sizes, degrees of freedom and *P* value noted<br>*Give P values as exact values whenever suitable.*
☒	☐	For Bayesian analysis, information on the choice of priors and Markov chain Monte Carlo settings
☐	☒	For hierarchical and complex designs, identification of the appropriate level for tests and full reporting of outcomes
☐	☒	Estimates of effect sizes (e.g. Cohen's *d*, Pearson's *r*), indicating how they were calculated

Our web collection on statistics for biologists contains articles on many of the points above.

Software and code

Policy information about availability of computer code

Data collection	MotionMonitor 9.31
Data analysis	MATLAB 2020b; FSL 6.0; Advanced Normalization Tools (ANTs) 2.3.4

For manuscripts utilizing custom algorithms or software that are central to the research but not yet described in published literature, software must be made available to editors and reviewers. We strongly encourage code deposition in a community repository (e.g. GitHub). See the Nature Portfolio guidelines for submitting code & software for further information.

Data

Policy information about availability of data

All manuscripts must include a data availability statement. This statement should provide the following information, where applicable:

- Accession codes, unique identifiers, or web links for publicly available datasets
- A description of any restrictions on data availability
- For clinical datasets or third party data, please ensure that the statement adheres to our policy

The clinical and neuroimaging data in this study are openly available in the Parkinson’s disease biomarker program (https://pdbp.ninds.nih.gov). Accelerometer data can be requested by contacting the corresponding authors.

Research involving human participants, their data, or biological material

Policy information about studies with human participants or human data. See also policy information about sex, gender (identity/presentation), and sexual orientation and race, ethnicity and racism.

Reporting on sex and gender	Participants of both biological sexes were included. Participant's self-reported biological sex. Statistical analyses included sex as a biological variable. Gender was not used as part of the analysis in this study.
Reporting on race, ethnicity, or other socially relevant groupings	This work did not implement socially constructed categorization variables.
Population characteristics	This manuscript included populations of Parkinson's disease, progressive supranuclear palsy, multiple systems atrophy, and healthy aging controls. The age and disease duration are described in Table 1 of this manuscript.
Recruitment	Subjects were referred from and diagnosed by movement disorder specialists at the University of Florida (UF) and Northwestern University (NU).
Ethics oversight	Institutional Review Boards - University of Florida and Northwestern University

Note that full information on the approval of the study protocol must also be provided in the manuscript.

Field-specific reporting

Please select the one below that is the best fit for your research. If you are not sure, read the appropriate sections before making your selection.

☒ Life sciences ☐ Behavioural & social sciences ☐ Ecological, evolutionary & environmental sciences

For a reference copy of the document with all sections, see nature.com/documents/nr-reporting-summary-flat.pdf

Life sciences study design

All studies must disclose on these points even when the disclosure is negative.

Sample size	Sample size for PPT neuroimaging was determined based on power calculations from effects determined in prior work (e.g. Burciu et al., 2016; Ofori et al. 2016; Mitchell et al., 2022). Sample size for accelerometer portion of the study was also based on power calculations from prior work (Wilkes et al., 2020).
Data exclusions	Data were excluded if a participant did not complete all required study measures.
Replication	Analyses were performed and verified by two separate individuals using the same workflows.
Randomization	Participants were allocated into groups based on diagnosis (e.g. Parkinson's disease).
Blinding	Random assignment was not possible, as experimental manipulation was performed. Individuals with Parkinsonism are discernable to study staff, so it is not possible to blind their diagnosis. All analyses were performed using automated workflows, so it was not possible to bias results.

Reporting for specific materials, systems and methods

We require information from authors about some types of materials, experimental systems and methods used in many studies. Here, indicate whether each material, system or method listed is relevant to your study. If you are not sure if a list item applies to your research, read the appropriate section before selecting a response.

Materials & experimental systems

n/a	Involved in the study	
☒	☐	Antibodies
☒	☐	Eukaryotic cell lines
☒	☐	Palaeontology and archaeology
☒	☐	Animals and other organisms
☒	☐	Clinical data
☒	☐	Dual use research of concern
☒	☐	Plants

Methods

n/a	Involved in the study	
☒	☐	ChIP-seq
☒	☐	Flow cytometry
☐	☒	MRI-based neuroimaging

Magnetic resonance imaging

Experimental design

Design type	Diffusion MRI
Design specifications	Diffusion MRI were gathered at baseline visit and at one-year followup.
Behavioral performance measures	Diffusion MRI is structural, and no performance measures are collected in relation to it.

Acquisition

Imaging type(s)	T1-weighted anatomical; Diffusion MRI
Field strength	3
Sequence & imaging parameters	T1-weighted images (repetition time: 2000 ms, echo time: 2.99 ms, flip angle: 8°, TI=1010 ms, GRAPPA factor=2, 0.8 mm isotropic voxels, bandwidth: 240 Hz/pixel) were acquired with a three-dimensional (3D) magnetization-prepared 180-degree radio-frequency pulses and rapid gradient-echo (MP-RAGE) sequence in 208 contiguous sagittal slices.<br><br>Single-shell diffusion MRI images (repetition time: 6400 ms, echo time: 58 ms, flip angle: 90°, field of view: 256 x 256 mm, resolution: 2 mm isotropic, 64 diffusion gradient directions, b-values: 5 x 0, and 64 x 1000 s/mm2, 69 axial slices, bandwidth: 2442 Hz/pixel, total acquisition time: 7 minutes 41 seconds) were acquired for free-water imaging.
Area of acquisition	whole brain

Diffusion MRI ☒ Used ☐ Not used

Parameters: 5 b=0 s/mm2 and 64 b=1000 s/mm2

Preprocessing

Preprocessing software	FSL 6.0 - BET, EDDY<br>MATLAB - free water estimation and elimination
Normalization	Advanced Normalisation Tools (ANTs) - nonlinear
Normalization template	Fractional anisotropy template, based off of MNI 152 template in standard space.
Noise and artifact removal	Eddy current and motion correction through FSL
Volume censoring	FSL - EDDY

Statistical modeling & inference

Model type and settings	Predicting PPT from neuroimaging: backward linear regression
Effect(s) tested	PPT score on the Both hands task at visit 2 was predicted using 36 neuroimaging metrics from visit1.

Specify type of analysis: ☐ Whole brain ☒ ROI-based ☐ Both

Anatomical location(s): There were eighteen regions or tracts of interest. These were primary motor cortex (M1), supplementary motor area (SMA), pre-supplementary motor area (preSMA), superior frontal gyrus (SFG), middle frontal gyrus (MFG), inferior parietal lobule (IPL), supramarginal gyrus (SMG), caudate, putamen, subthalamic nucleus (STN), posterior substantia nigra (pSN), pedunculopontine nucleus (PPN), and dentate nucleus of the cerebellum.

A total of five white matter tracts were investigated. Three white matter tracts were from the sensorimotor area tract template (SMATT; Archer et al., 2018) which included descending sensorimotor tracts from M1, SMA, and preSMA. One tract from cerebellar probabilistic white matter atlas (van Baarsen et al., 2016), the superior cerebellar peduncle (SCP). One tract was from the Parkinson's disease region of interest template (Archer et al., 2019), the nigrostriatal tract.

Statistic type for inference<br>(See Eklund et al. 2016)	In this manuscript, we did not perform direct group-wise comparisons of neuroimaging variables. Rather, these measures were used for backward linear regression in order to predict Purdue Pegboard score at one-year followup.
Correction	FDR

Models & analysis

n/a	Involved in the study
☒	☐ Functional and/or effective connectivity
☒	☐ Graph analysis
☐	☒ Multivariate modeling or predictive analysis

Multivariate modeling and predictive analysis

Backward linear regression of imaging variables at visit 1 was used predict Purdue Pegboard score at visit 2.

For imaging predictors, there were eighteen regions or tracts of interest, and the FW and FAt values for each were included. Thirteen grey matter regions were included from the Parkinson's disease region of interest template validated in previous work by our group (Archer et al., 2019). These were primary motor cortex (M1), supplementary motor area (SMA), pre-supplementary motor area (preSMA), superior frontal gyrus (SFG), middle frontal gyrus (MFG), inferior parietal lobule (IPL), supramarginal gyrus (SMG), caudate, putamen, subthalamic nucleus (STN), posterior substantia nigra (pSN), pedunculopontine nucleus (PPN), and dentate nucleus of the cerebellum. A total of five white matter tracts were investigated. Three white matter tracts were from the sensorimotor area tract template (SMATT; Archer et al., 2018) which included descending sensorimotor tracts from M1, SMA, and preSMA. One tract from cerebellar probabilistic white matter atlas (van Baarsen et al., 2016), the superior cerebellar peduncle (SCP). One tract was from the Parkinson's disease region of interest template (Archer et al., 2019), the nigrostriatal tract.
